# Could Mineralocorticoids Play a Role in the Pathophysiology of Open Angle Glaucoma?

**DOI:** 10.1155/2012/196418

**Published:** 2011-08-25

**Authors:** Christian Albrecht May

**Affiliations:** Department of Anatomy, Faculty of Medicine Carl Gustav Carus, TU Dresden, 01307 Dresden, Germany

## Abstract

Since the pathomechanisms of primary open angle glaucoma are still not defined, different aspects related to this topic have to be discussed and further investigated. Possible candidates are the mineralocorticoids, which are known to lower intraocular pressure. A data search and personal investigations assume a limited role of mineralocorticoids for the development of glaucoma. Specific experiments for a final conclusion are, however, not yet performed.

## 1. The Present Defined Risk Factors for Open Angle Glaucoma Are Poor

Glaucoma comprises a number of different pathomechanisms leading to a specific degeneration of the retinal ganglion cells and changes in the optic nerve head. Primary open angle glaucoma is the most common form and affects about 1% of the western population.

To date, the only defined risk factors for the development of primary open angle glaucoma (POAG) are age and elevated intraocular pressure (IOP). Both factors are complex and their precise role and regulation are not known. Patho-morphological correlations discussed for the elevated IOP are the appearance of “plaque-like extracellular material” in the human trabecular meshwork [1, 2], “empty spaces/giant vacuoles” in the juxtacanalicular region next to Schlemm's canal [3–5], and the size of Schlemm's canal itself [6, 7]. The active role of TM cells in this regulative process was considered due to their contractile properties [8, 9] and due to intracellular volume regulation [10–12]. Interestingly, the increased production of aqueous humour alone seems not to be responsible for elevated intraocular pressure, although a number of therapies modify this input.

In recent years, a broader understanding of intracellular volume regulation was gained by the description and investigation of specific ion channels and their molecular regulation. In this context, corticoid hormones played a crucial role [13, 14]. This paper tries to bring this knowledge forward to glaucoma pathophysiology.

## 2. Glucocorticoids Are Known for Their Ocular Hypertensive Property

Early investigations of steroid hormone function in the eye were lead by clinical observations of IOP elevation in one-third of the population after topical cortisone treatment [15, 16]. Persisting ocular hypertension can lead to a specific type of open angle glaucoma, the “cortisone-induced” glaucoma [17, 18] with a typical morphological appearance [19, 20]. Interestingly, systemic elevation of cortisone can slightly increase IOP but does not lead to a higher risk of glaucoma development [21]. Therefore, local mechanisms seem to play a crucial role. One of them is the 11*β*-hydroxysteroid dehydrogenase (HSD) consisting of two isozymes with distinct different functions. HSD1 is the key enzyme for activation of cortisone; HSD2 leads to inactivation of cortisone in specific tissues with aldosterone receptors which could also be activated by cortisone. To postulate an effect of cortisone, HSD1 for activation and the glucocorticoid receptor (GR) should both be present. In the trabecular meshwork GR and HSD1 were described originally [22], but subsequent studies only confirmed the presence of GR [23, 24]. From a functional point of view, glucocorticoids lead to an intracellular volume increase in trabecular meshwork cells [25–27] and modify the extracellular matrix production [28, 29]. Most surprisingly, one of the extracellular matrix proteins affected is elevated in all human glaucomatous donor eyes [30, 31], but physiological studies recently questioned its role for elevation of trabecular meshwork resistance and IOP [32, 33]. Thus the cellular volume increase effect of cortisone has the best evidence to be of pathophysiologic relevance for IOP increase at present.

## 3. Mineralocorticoids Have Some Effects in the Eye

For a long time, a second group of corticoid hormones, the mineralocorticoids, were not considered to play any significant function in the eye [34]. However, early investigations mentioned that the aldosterone-antagonist spironolacton led to a decrease of intraocular pressure in glaucomatous patients [35]. Mirshahi and coworkers were the first to describe mineralocorticoid hormone receptors (MR) in the retina and all epithelial cells of the eye [36, 37]. To consider specific aldosterone function, the presence of HSD2 next to the MR is necessary. The presence of MR and HSD2 in the trabecular meshwork is described controversially [22–24]. Mineralocorticoid effects are thought to be mediated by epithelial sodium channels (EnaC) [38, 39], which are also present at numerous places in the anterior eye segment [40–43]. These channels might serve two different functions: one is fluid secretion from the ciliary epithelium (increase of aqueous humour formation), and the other is regulation of the trabecular meshwork resistance by volume regulation of the trabecular meshwork cells. The first is the most widely suggested mechanism for aldosterone [44] since a consistent presence of MR and a strong evidence for the presence of HSD2 are reported in ciliary epithelium cells [22–24]. If the trabecular meshwork is also a target tissue for aldosteron remains to be determined.

## 4. Can Mouse Eye Models Help Concerning Mineralocorticoid Effects?

A number of mouse models were established to study mineralocorticoid effects but no data exists about the eyes of these animals. The existing genetically altered mice show either an overexpression of the MR [44–46], a knockout of HSD2 [47], or alterations of the ENaC ion channels (Liddle's syndrome) [48, 49].

**Figure 1:**
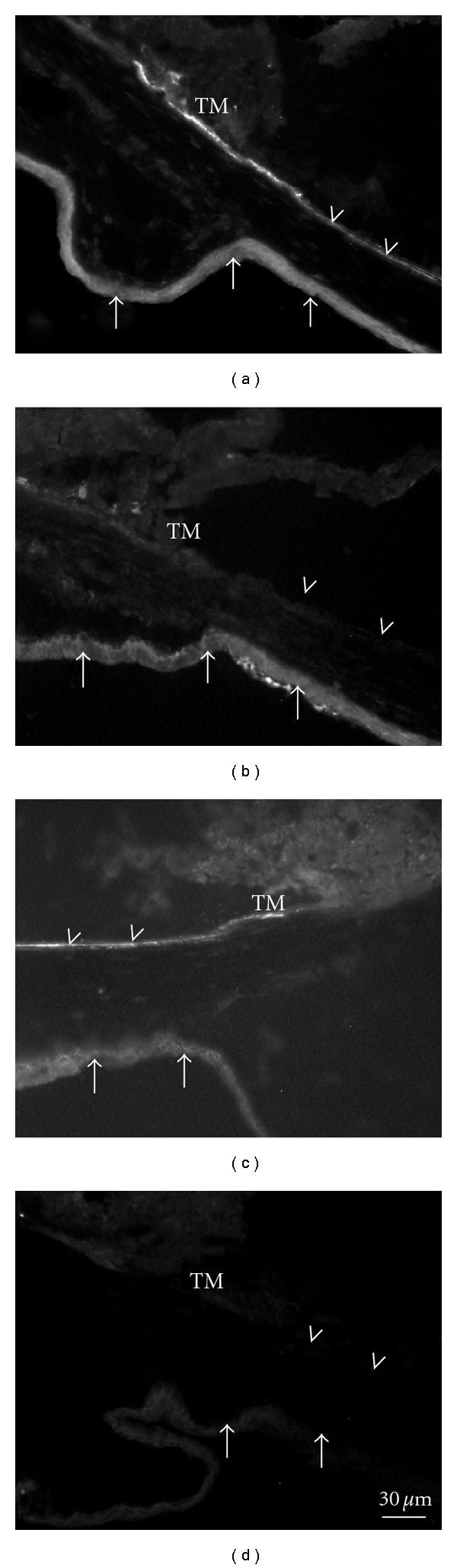
Immunohistochemical staining of the mouse anterior segment with antibodies against *α*-ENaC (a), *β*-ENaC (b), and *γ*-ENaC (c), immune sera were kindly provided by Bernard Rossier and Christoph Korbmacher, and without primary antibody (d). Note the intense staining of the trabecular meshwork (TM) and corneal endothelium (arrowheads) with *α*- and *γ*-ENaC, but not with *β*-ENaC. Arrows: conjunctiva and cornea epithelium. The conjunctiva did not show staining with antibodies against *γ*-ENaC (arrows in (c)).

Unfortunately, there is no data in mouse eyes for the presence and distribution of MR and HSD2. Personal investigations on the distribution of ENaC in the mouse anterior eye segment showed intense staining for *α*- and *γ*-EnaC, but no staining for *β*-EnaC in the trabecular meshwork, while the ciliary epithelium showed only a weak staining reaction (Figure 1).

Overexpression of MR was induced in B6D2 animals. During embryogenesis, MR overexpression led to massive changes in the anterior eye chamber due to epidermal atrophy in these nonviable puppets [46]. Unfortunately, these animals have a DBA/2J background leading to changes in the chamber angle beginning at 3 months of age. Personal investigations on the eyes of 6-months-old transgenic animals (P1.hMR and P2.hMR from [44, 45]) show massive synechiae of the iris, atrophy of the ciliary body, strong pigmentation of the chamber angle, and loss of retinal ganglion cells. These findings match with findings observed in other DBA strains [50–52]. Specific mineralocorticoid related changes could not be observed. Knockout of HSD2 was performed in C57/Bl6 mice.

The mouse model established for Liddle's syndrome has an altered *β*-EnaC subunit. There seems to be no effect of aldosteron on the *α*-EnaC subunit in these animals [53]. Personal investigations on eyes of these animals revealed a normal morphology. Schlemm's canal was widely open, trabecular meshwork cells were not swollen, abnormalities in the anterior and posterior eye segments could not be detected. Since the normal mouse eye does not express the *β*-EnaC subunit in the trabecular meshwork and inner eye surfaces, these results are not surprising.

Concluding, at this stage of research mouse models do not help to answer questions related to the role of mineralocorticoids in the eye.

## 5. Specific Mineralocorticoid Dysfunctions Also Exist in the Human: How about Ocular Pathology in These Patients?

A number of conditions are known in the human associated with mineralocorticoid dysfunction. A relation to ocular pathologies was tested.

Hyperaldosteronism is a common, but rarely diagnosed condition (estimated 1.5–3.5% of the entire population in Germany [54]). The induced high blood pressure can affect the eye but not in a glaucoma-specific way. There are no functional changes in the eye related to this general condition. It remains to be determined if these patients show any association to glaucoma.

Apparent mineralocorticoid excess is a condition with lack of HSD2 and subsequently increased activation of MR [55]. There is no report in the literature that any of the diagnosed patients suffered from either elevated IOP or glaucoma.

Pseudohypoaldosteronism type 1 is related to a reduction of alpha ENaC function [56, 57]. A communication with Prof. Hanukoglu (Tel Aviv) revealed that these persons do not complain of any specific eye symptoms. An extended ocular examination of his oldest patient at that time (19 years old) showed normal intraocular pressure (17 mmHg), a normal anterior chamber including the chamber angle, and a normal OCT of the nerve fibers in the retina. The only finding was a slightly increased corneal thickness (570 *μ*m). If this finding is related to a reduced function of the corneal endothelium remains to be determined.

## 6. Is There Any Mineralocorticoid Input to Glaucoma Pathophysiology at Present?

Systemic application of mineralocorticoids to glaucoma patients shows no changes in the IOP in most of the cases [58, 59]. However, single individuals react with a high increase in intraocular pressure [58]. Unfortunately, these “mineralocorticoid-sensitive” persons are not further characterized. They could constitute a new subgroup of ocular hypertension or glaucoma patients, but more clinical data has to be collected to define these persons.

The lack of general agonist effects combined with the mild IOP decrease of antagonists [35] points to a possible role of mineralocorticoids for glaucoma therapy but not for general glaucoma pathophysiology. The narrowed role is also supported by the negative findings in the animal models and the various human conditions described above. The therapeutic effect of mineralocorticoid antagonists seems mainly mediated by a decrease of aqueous humour formation [36]. If there is some effect on the outflow pathway remains open.

One additional aspect of mineralocorticoid function is venoconstriction and thus an increase in postcapillary pressure [60, 61]. Venoconstriction could also be of relevance for elevated intraocular pressure as known from rat glaucoma models [62]. If mineralocorticoid mediated venoconstriction is also present in limbal veins remains to be determined. A different venous sensitivity could be a criterion for the above hypothesized mineralocorticoid-sensitive subgroup of humans.

## 7. Conclusion

**Figure 2:**
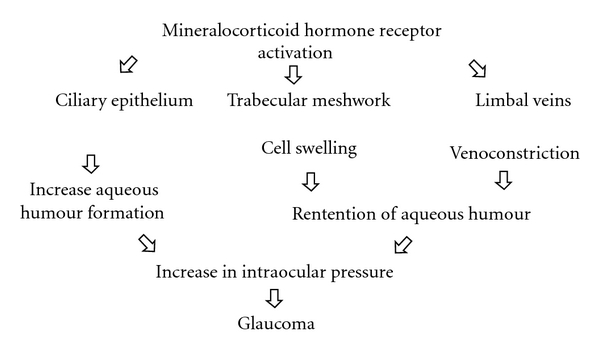
Proposed mechanisms by which mineralocorticoids play a role in glaucoma.

The proposed mechanisms by which mineralocorticoids play a role in glaucoma are summarized in Figure 2. While there is some evidence that the ciliary epithelium is affected by mineralocorticoids, the role of the trabecular meshwork cells and of the limbal veins remains to be determined. Hopefully this paper attracts more scientists and clinicians for further research in the area of mineralocorticoids with respect to the pathogenesis of glaucoma.

## References

[1] Rohen JW, Witmer R (1972). Electron microscopic studies on the trabecular meshwork in glaucoma simplex. *Albrecht von Graefe’s Archive for Clinical and Experimental Ophthalmology*.

[2] Rohen JW, Lutjen-Drecoll E, Flugel C, Meyer M, Grierson I (1993). Ultrastructure of the trabecular meshwork in untreated cases of primary open-angle glaucoma (POAG). *Experimental Eye Research*.

[3] Fink AI, Felix MD, Fletcher RC (1978). The anatomic basis for glaucoma. *Annals of Ophthalmology*.

[4] Ye W, Gong H, Sit A, Johnson M, Freddo TF (1997). Interendothelial junctions in normal human schlemm’s canal respond to changes in pressure. *Investigative Ophthalmology and Visual Science*.

[5] Brilakis HS, Johnson DH (2001). Giant vacuole survival time and implications for aqueous humor outflow. *Journal of Glaucoma*.

[6] Moses RA (1981). The conventional outflow resistances. *American Journal of Ophthalmology*.

[7] Van Buskirk EM (1982). Anatomic correlates of changing aqueous outflow facility in excised human eyes. *Investigative Ophthalmology and Visual Science*.

[8] Grierson I, Rahi AHS (1979). Microfilaments in the cells of the human trabecular meshwork. *British Journal of Ophthalmology*.

[9] Lepple-Wienhues A, Stahl F, Wiederholt M (1991). Differential smooth muscle-like contractile properties of trabecular meshwork and ciliary muscle. *Experimental Eye Research*.

[10] Putney LK, Brandt JD, O’Donnell ME (1999). Na-K-Cl cotransport in normal and glaucomatous human trabecular meshwork cells. *Investigative Ophthalmology and Visual Science*.

[11] Mitchell CH, Fleischhauer JC, Daniel Stamer W, Peterson-Yantorno K, Civan MM (2002). Human trabecular meshwork cell volume regulation. *American Journal of Physiology*.

[12] Soto D, Comes N, Ferrer E (2004). Modulation of aqueous humor outflow by ionic mechanisms involved in trabecular meshwork cell volume regulation. *Investigative Ophthalmology and Visual Science*.

[13] Fillon S, Wärntges S, Matskevitch J (2001). Serum- and glucocorticoid-dependent kinase, cell volume, and the regulation of epithelial transport. *Comparative Biochemistry and Physiology*.

[14] Harvey BJ, Alzamora R, Healy V, Renard C, Doolan CM (2002). Rapid responses to steroid hormones: from frog skin to human colon. A homage to Hans Ussing. *Biochimica et Biophysica Acta*.

[15] Armaly MF (1965). Statistical attributes of the steroid hypertensive response in the clinically normal eye. I.The demonstration of three levels of response. *Investigative Ophthalmology*.

[16] Armaly MF, Becker B (1965). Intraocular pressure response to topical corticosteroids. *Federation Proceedings*.

[17] Miller SJ (1965). Steroid glaucoma. *Transactions of the Ophthalmological Societies of the United Kingdom*.

[18] Fritz A (1965). Insufficiency of aqueous suction by the circulating blood and cortisone-induced glaucoma. *Bulletin de la Societe Belge d’Ophtalmologie*.

[19] Rohen JW (1973). Fine structural changes in the trabecular meshwork of the human eye in different forms of glaucoma. *Klinische Monatsblatter fur Augenheilkunde*.

[20] Johnson D, Gottanka J, Flugel C, Hoffmann F, Futa R, Lutjen-Drecoll E (1997). Ultrastructural changes in the trabecular meshwork of human eyes treated with corticosteroids. *Archives of Ophthalmology*.

[21] Huschle OK, Jonas JB, Koniszewski G, Buchfelder M, Fahlbusch R (1990). Glaucom und das zentrale hypothalamisch-hypophysäre Cushing syndrom. *Fortschritte der Ophthalmologie*.

[22] Stokes J, Noble J, Brett L (2000). Distribution of glucocorticoid and mineralocorticoid receptors and 11beta-hydroxysteroid dehydrogenases in human and rat ocular tissues. *Investigative Ophthalmology and Visual Science*.

[23] Suzuki T, Sasano H, Kaneko C, Ogawa S, Darnel AD, Krozowski ZS (2001). Immunohistochemical distribution of 11*β*-hydroxysteroid dehydrogenase in human eye. *Molecular and Cellular Endocrinology*.

[24] Rauz S, Walker EA, Shackleton CH, Hewison M, Murray PI, Stewart PM (2001). Expression and putative role of 11 beta-hydroxysteroid dehydrogenase isozymes within the human eye. *Investigative Ophthalmology and Visual Science*.

[25] Tripathi BJ, Tripathi RC, Swift HH (1989). Hydrocortisone-induced DNA endoreplication in human trabecular cells in vitro. *Experimental Eye Research*.

[26] Clark AF, Wilson K, McCartney MD, Miggans ST, Kunkle M, Howe W (1994). Glucocorticoid-induced formation of cross-linked actin networks in cultured human trabecular meshwork cells. *Investigative Ophthalmology and Visual Science*.

[27] Wilson K, McCartney MD, Miggans ST, Clark AF (1993). Dexamethasone induced ultrastructural changes in cultured human trabecular meshwork cells. *Current Eye Research*.

[28] Stone EM, Fingert JH, Alward WLM (1997). Identification of a gene that causes primary open angle glaucoma. *Science*.

[29] Tamm ER (2002). Myocilin and glaucoma: facts and ideas. *Progress in Retinal and Eye Research*.

[30] Lutjen-Drecoll E, May CA, Polansky JR, Johnson DH, Bloemendal H, Nguyen TD (1998). Localization of the stress proteins alpha B-crystallin and trabecular meshwork inducible glucocorticoid response protein in normal and glaucomatous trabecular meshwork. *Investigative Ophthalmology and Visual Science*.

[31] Tamm ER, Russell P, Epstein DL, Johnson DH, Piatigorsky J (1999). Modulation of myocilin/TIGR expression in human trabecular meshwork. *Investigative Ophthalmology and Visual Science*.

[32] Gould DB, Miceli-Libby L, Savinova OV (2004). Genetically increasing Myoc expression supports a necessary pathologic role of abnormal proteins in glaucoma. *Molecular and Cellular Biology*.

[33] Zillig M, Wurm A, Grehn FJ, Russell P, Tamm ER (2005). Overexpression and properties of wild-type and Tyr437His mutated myocilin in the eyes of transgenic mice. *Investigative Ophthalmology and Visual Science*.

[34] Kass MA, Sears ML (1977). Hormonal regulation of intraocular pressure. *Survey of Ophthalmology*.

[35] Witzmann R (1980). The effect of spironolactone on intraocular pressure in glaucoma patients. *Klinische Monatsblatter fur Augenheilkunde*.

[36] Mirshahi M, Nicolas C, Mirshahi A (1996). The mineralocorticoid hormone receptor and action in the eye. *Biochemical and Biophysical Research Communications*.

[37] Mirshahi M, Mirshahi A, Sedighian R, Hecquet C, Faure JP, Agarwal MK (1997). Immunschemical demonstration of the mineualocauticoid receptor in ocular tissues. *Neuroendocrinology*.

[38] Mirshahi M, Mirshahi S, Golestaneh N (2001). Mineralocorticoid hormone signaling regulates the ’epithelial sodium channel’ in fibroblasts from human cornea. *Ophthalmic Research*.

[39] Golestaneh N, Picaud S, Mirshahi M (2002). The mineralocorticoid receptor in rodent retina: ontogeny and molecular identity. *Molecular Vision*.

[40] Civan MM, Peterson-Yantorno K, Sánchez-Torres J, Coca-Prados M (1997). Potential contribution of epithelial Na+ channel to net secretion of aqueous humor. *Journal of Experimental Zoology*.

[41] Mirshahi M, Nicolas C, Mirshahi S, Golestaneh N, D’Hermies F, Agarwal MK (1999). Immunochemical analysis of the sodium channel in rodent and human eye. *Experimental Eye Research*.

[42] Rauz S, Walker EA, Hughes SV (2003). Serum- and glucocorticoid-regulated kinase isoform-1 and epithelial sodium channel subunits in human ocular ciliary epithelium. *Investigative Ophthalmology and Visual Science*.

[43] Rauz S, Walker EA, Murray PI, Stewart PM (2003). Expression and distribution of the serum and glucocorticoid regulated kinase and the epithelial sodium channel subunits in the human cornea. *Experimental Eye Research*.

[44] Le Menuet DL, Viengchareun S, Penfornis P, Walker F, Zennaro MC, Lombès M (2000). Targeted oncogenesis reveals a distinct tissue-specific utilization of alternative promoters of the human mineralocorticoid receptor gene in transgenic mice. *Journal of Biological Chemistry*.

[45] Le Menuet D, Zennaro MC, Viengchareun S, Lombès M (2000). Transgenic mouse models to study human mineralocorticoid receptor function in vivo. *Kidney International*.

[46] Marie YS, Toulon A, Paus R (2007). Targeted skin overexpression of the mineralocorticoid receptor in mice causes epidermal atrophy, premature skin barrier formation, eye abnormalities, and alopecia. *American Journal of Pathology*.

[47] Kotelevtsev Y, Brown RW, Fleming S (1999). Hypertension in mice lacking 11*β*-hydroxysteroid dehydrogenase type 2. *Journal of Clinical Investigation*.

[48] Pradervand S, Vandewalle A, Bens M (2003). Dysfunction of the epithelial sodium channel expressed in the kidney of a mouse model for Liddle syndrome. *Journal of the American Society of Nephrology*.

[49] Dahlmann A, Pradervand S, Hummler E, Rossier BC, Frindt G, Palmer LG (2003). Mineralocorticoid regulation of epithelial Na+ channels is maintained in a mouse model of Liddle’s syndrome. *American Journal of Physiology*.

[50] John SWM, Smith RS, Savinova OV (1998). Essential iris atrophy, pigment dispersion, and glaucoma in DBA/2J mice. *Investigative Ophthalmology and Visual Science*.

[51] Chang B, Smith RS, Hawes NL (1999). Interacting loci cause severe iris atrophy and glaucoma in DBA/2J mice. *Nature Genetics*.

[52] Bayer AU, Neuhardt T, May AC (2001). Retinal morphology and ERG response in the DBA/2NNia mouse model of angle-closure glaucoma. *Investigative Ophthalmology and Visual Science*.

[53] Bertog M, Cuffe JE, Pradervand S (2008). Aldosterone responsiveness of the epithelial sodium channel (ENaC) in colon is increased in a mouse model for Liddle’s syndrome. *Journal of Physiology*.

[54] Schirpenbach C, Segmiller F, Diederich S (2009). The diagnosis and treatment of primary hyperaldosteronism in Germany - Results on 555 patients from the German Conn Registry. *Deutsches Arzteblatt*.

[55] Stewart PM, Corrie JET, Shackleton CHL, Edwards CRW (1988). Syndrome of apparent mineralocorticoid excess. A defect in the cortisol-cortisone shuttle. *Journal of Clinical Investigation*.

[56] Hanukoglu A, Hanukoglu I (2010). Clinical improvement in patients with autosomal recessive pseudohypoaldosteronism and the necessity for salt supplementation. *Clinical and Experimental Nephrology*.

[57] Hanukoglu A, Edelheit O, Shriki Y, Gizewska M, Dascal N, Hanukoglu I (2008). Renin-aldosterone response, urinary Na/K ratio and growth in pseudohypoaldosteronism patients with mutations in epithelial sodium channel (ENaC) subunit genes. *Journal of Steroid Biochemistry and Molecular Biology*.

[58] Frenkel M, Krill AR (1964). Effects of two mineralocorticoids on ocular tension. *Archives of Ophthalmology*.

[59] Gugleta K, Orgül S, Stümpfig D, Dubler B, Flammer J (1999). Fludrocortisone in the treatment of systemic hypotension in primary open-angle glaucoma patients. *International Ophthalmology*.

[60] Fink GD, Johnson RJ, Galligan JJ (2000). Mechanisms of increased venous smooth muscle tone in desoxycorticosterone acetate-salt hypertension. *Hypertension*.

[61] Li L, Watts SW, Banes AK, Galligan JJ, Fink GD, Chen AF (2003). NADPH oxidase-derived superoxide augments endothelin-1-induced venoconstriction in mineralocorticoid hypertension. *Hypertension*.

[62] Morrison JC, Johnson E, Cepurna WO (2008). Rat models for glaucoma research. *Progress in Brain Research*.

